# Multiple transfused thalassemia major: Ocular manifestations in a hospital-based population

**DOI:** 10.4103/0301-4738.60083

**Published:** 2010

**Authors:** Rashi Taneja, Pankaj Malik, Mamta Sharma, Mahesh C Agarwal

**Affiliations:** Departments of Ophthalmology and Pediatrics, Din Dayal Upadhyay Hospital, Hari Nagar, New Delhi - 110 064, India

**Keywords:** Desferrioxamine, deferriprone, iron overload, thalassemia

## Abstract

**Purpose:**

To study the ocular manifestations in multiple transfused beta-thalassemia major patients and assess the ocular side-effects of iron chelating agents.

**Materials and Methods:**

In this prospective observational study, 45 multiple transfused beta-thalassemia major children between six months and 21 years of age were enrolled and assigned groups according to the treatment regimens suggested. Group A received only blood transfusions, Group B blood transfusions with subcutaneous desferrioxamine, Group C blood transfusions with desferrioxamine and oral deferriprone and Group D blood transfusions with deferriprone. Ocular status at the time of enrolment was documented. Subjects were observed quarterly for one year for changes in ocular status arising due to the disease process and due to iron chelation therapy. Children with hemoglobinopathies other than beta-thalassemia major, congenital ocular anomalies and anemia due to other causes were excluded.

**Results:**

Ocular involvement was observed in 58% of patients. Lenticular opacities were the most common ocular finding (44%), followed by decreased visual acuity (33%). An increased occurrence of ocular changes was observed with increase of serum ferritin and serum iron levels as well as with higher number of blood transfusions received. Desferrioxamine seemed to have a protective influence on retinal pigment epithelium (RPE) mottling. Occurrence of lenticular opacities and RPE degeneration correlated positively with use of desferrioxamine and deferriprone respectively. Follow-up of patients for one year did not reveal any change in ocular status.

**Conclusion:**

Regular ocular examinations can aid in preventing, delaying or ameliorating the ocular complications of thalassemia.

Thalassemias are the most common single gene disorder worldwide.[1] Mutations involving the beta globin gene in beta-thalassemia cause disruption in red blood cell maturation leading to ineffective erythropoiesis and multi-system involvement. Multiple/repeated blood transfusions lead to siderosis.[2,3] Adverse ocular changes may occur as a result of the disease itself or as side-effects of iron chelators[4–8] and include cataract, optic neuropathy, retinal pigment epithelial (RPE) degeneration, RPE mottling, retinal venous tortuosity, vitreoretinal hemorrhages and obliteration of iris pattern [Fig. 1]. Desferrioxamine and deferriprone, which are used to avoid systemic complications of siderosis cause chelation of metals such as iron, copper, zinc, cobalt and nickel in the retina. These metals are essential for normal retinal function.[6,8–11] The aim of the study was to know the ocular manifestations in multiple transfused beta-thalassemia major patients and assess the ocular side-effects of iron-chelating agents.

**Figure 1:**
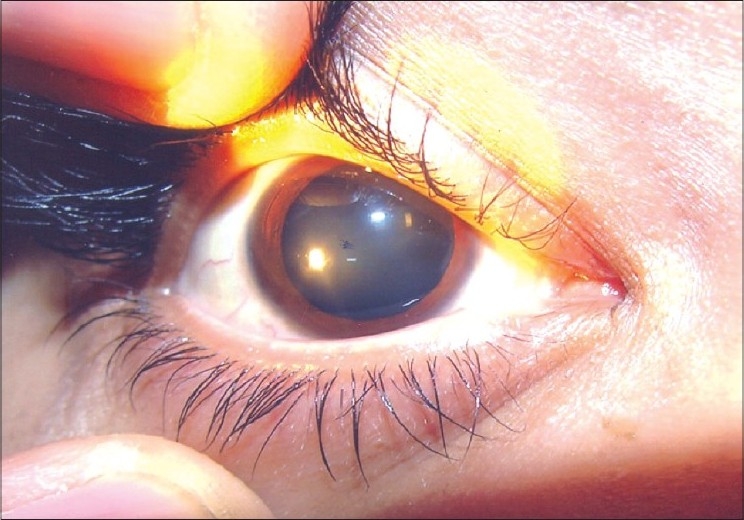
Loss of iris pattern in a child suffering from thalassemia major

## Materials and Methods

In this prospective observational study lasting for one year, 45 children with beta-thalassemia major attending the thalassemia clinic of the pediatric department of the hospital were enrolled. All patients were informed about the nature of the study and an informed consent for participation obtained. In case of minors, consent was taken from their parents. Subjects with hemoglobinopathies other than beta-thalassemia major, anemias due to other causes and with congenital ocular anomalies were excluded from the study. The diagnosis of beta-thalassemia major was confirmed by clinical, hematological and electrophoretic studies. All patients received scheduled blood transfusions at three to four-week intervals so as to maintain a hemoglobin level ≥9-9.5 gm/dl.

**Figure 2:**
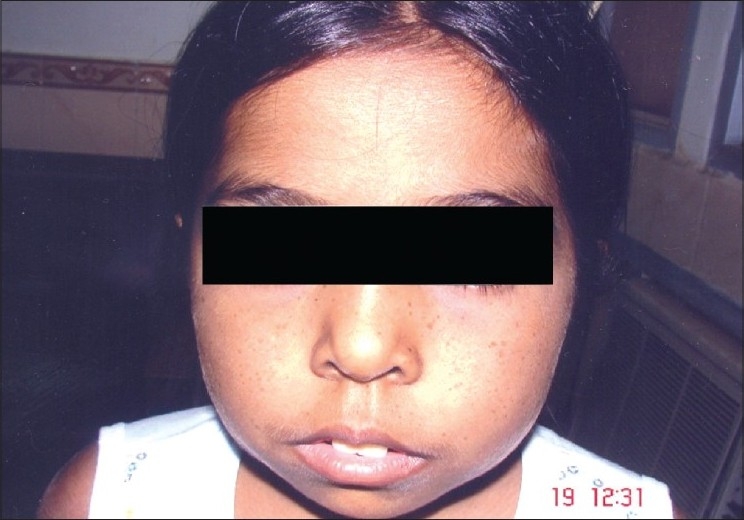
Frontal bossing and prominent maxilla (thalassemic facies) in a child suffering from thalassemia major

The patients were divided into four groups based on the thalassemia treatment regimes being followed by them at the time of presentation. Group A received only blood transfusions but no iron chelation therapy, Group B a combination regime of blood transfusions and subcutaneous desferrioxamine, Group C blood transfusions combined with subcutaneous desferrioxamine and oral deferriprone and Group D blood transfusions along with oral deferriprone. The first investigator who was a pediatrician, elicited a complete general history (including family history and details of previous blood transfusions and iron chelation therapy) and performed systemic examination, especially for presence of pallor, icterus, frontal bossing, prominent maxilla [Fig. 2], skin hyperpigmentation and hepatosplenomegaly. Laboratory investigations included baseline complete blood counts and serum ferritin and iron level estimations. The second investigator, who was an ophthalmologist elicited a complete ophthalmic history and performed ocular examination. Ocular examination included near and distance visual acuity assessment with and without glasses in all children using preferential looking test, picture cards and Snellen's charts as applicable, external examination with diffuse illumination, slit-lamp examination, direct and indirect ophthalmoscopy and fundus fluorescein angiography (FFA) in selected patients. The second investigator was masked to information obtained from the pediatric questionnaire. The first investigator too was masked to information on the ocular status of the patients. The patients were followed up at three-monthly intervals during which complete ocular assessment was done and progression in ocular changes, if any, noted. Serum ferritin levels were assessed at six-monthly intervals. Ocular findings at follow-up visits were correlated with the sex of the patient, number of blood transfusions received, serum iron levels, serum ferritin levels and doses and duration of desferrioxamine and deferriprone.

A *P* value less than 0.05 was considered statistically significant. Statistical analyses were performed using software SPSS v.10.0 (SPSS Inc., Chicago, Illinois, USA). For correlation of sex with lenticular opacities, RPE degeneration and RPE mottling, Chi^2^ test was used. Correlation of lens opacities, RPE degeneration and RPE mottling with serum ferritin levels, serum iron levels and average number of blood transfusions received was done by Pearson's correlational analysis with two-tailed *P* value <0.05 taken as significant. Correlation of lens opacities, RPE degeneration and RPE mottling with dose of desferrioxamine and of deferriprone in various groups too was obtained by Pearson's correlational analysis. Lens opacities, visual acuity, RPE degeneration, RPE mottling and retinal venous tortuosity were taken as ordered variables and assigned scores. Absence of lens opacities was assigned a score of 1, punctiform opacities in the posterior cortex were classified as mild opacities and graded as 2 while diffuse haziness extending in the posterior cortex and posterior subcapsular area was classified as moderate opacities and graded as 3. Absence of RPE degeneration was graded as 1 and its presence as 2. Doses of desferrioxamine and deferriprone, serum ferritin levels and serum iron levels were taken as continuous variables.

## Results

Table 1 shows the baseline characteristics of the study group. Of the 45 patients, 20 (44%) originated from Pakistan and 25 (56%) from India. Thirty-six (80%) patients were diagnosed to be suffering from beta-thalassemia within the first year of life, of whom 21 (58%) were diagnosed within six months of life. Four (9%) patients were diagnosed between the ages of one and two years, two (4%) between two and three years and three (7%) after the age of three. At first examination, 25 (56%) patients had already received between 100 to 300 blood transfusions. Mean number of transfusions received was 176.15 (range 1-401). Overall, of the 45 subjects, 19 (45%) received subcutaneous desferrioxamine. Total dose of prescribed desferrioxamine ranged from 52 to 3744 g (average = 939.42 g). Thirty-three (73%) subjects received oral deferriprone (dose range = 91.25-10220 g; average 2915.75 g). Average dose of deferriprone given to Group C was 3004 g while that given to Group D was 2858.4 g. Table 2 shows the changes in ocular status according to age. All patients above the age of 15 years showed some form of ocular changes, while in those under the age of five years, no ocular changes were seen, perhaps related to the duration of the disease. Overall, 26 (58%) patients had some ocular involvement.

**Table 1:** Group-wise baseline characteristics according to age and sex

	Group A (no iron chelators) (%)	Group B (desferrioxamine) (%)	Group C (desferrioxamine + deferriprone) (%)	Group D (deferriprone) (%)	Total (%)
Total subjects	6 (13)	6 (13)	13 (29)	20 (45)	45 (100)
Age group (years)					
≤5	5	-	-	2	7 (16)
6-10	-	3	5	7	15 (33)
11-15	1	2	3	7	13 (29)
16-20	-	1	4	4	9 (20)
>20	-	-	1	-	1 (2)
Sex					
Males	4	3	7	11	25 (56)
Females	2	3	6	9	20 (44)

**Table 2:** Changes in ocular status according to age

Age (years)	Number of subjects (%)	Ocular involvement (%)	Decreased visual acuity (%)	Lens opacities (%)	Fundus changes (%)
≤5	7	0	0	0	0
6-10	15	5	1	3	1
11-15	13	11	6	8	8
16-20	9	9	7	6	7
>20	1	1	1	1	1
Total (%)	45 (100)	26 (58)	15 (33)	18 (40)	17 (38)

**Figure 3:**
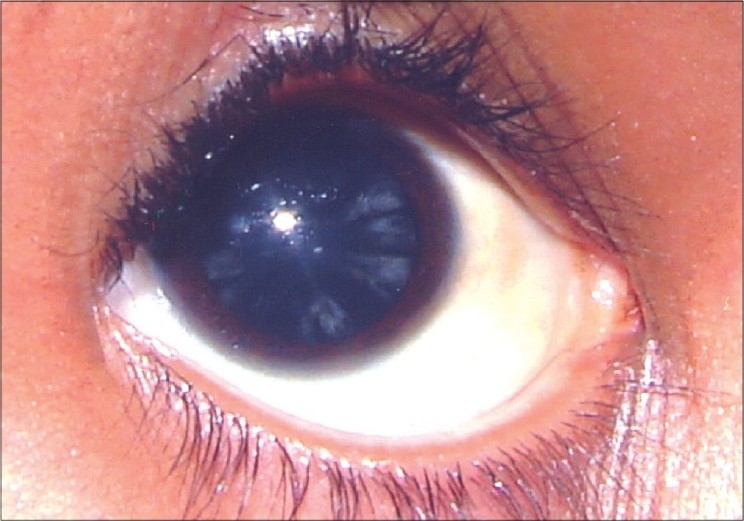
Lenticular opacities in a child suffering from thalassemia major

Table 3 shows ocular involvement in different groups of thalassemic patients. Thirty (67%) subjects had normal visual acuity. In all subjects, near visual acuity was normal. Lenticular opacities seen in thalassemic patients mainly comprised posterior subcapsular haze, streaks in posterior capsule and posterior cortical opacities [Fig. 3]. None of these opacities were in the visual axis and therefore none interfered with vision. Overall, 27 (60%) patients did not have lenticular opacities. None of the patients showed any abnormality on visual field testing. In those with decreased visual acuity, refractive error was found to be the cause and the vision was fully correctable with glasses.

**Table 3:** Group-wise comparisons of ocular involvement, visual acuity and fundus changes in beta-thalassemia subjects

	No. of subjects			
	
	Group A	Group B	Group C	Group D
Ocular involvement				
Present (58%)	1	4	8	13
Absent (42%)	5	2	5	7
Visual acuity				
Normal (67%)	5	3	8	14
Decreased (33%)	1	3	5	6
Lens opacities				
Present (40%)	1	3	8	6
Absent (60%)	5	3	5	14
Fundus changes				
RPE* degeneration (31%)	0	1	4	9
RPE mottling (9%)	1	0	1	2
Venous tortuosity (11%)	1	1	1	2
Disc hyperemia (7%)	1	0	1	1
Increased cup-disc ratio (4%)	0	0	1	1
No changes	5	5	9	9

* -Retinalpigmentepithelium

Average serum iron level in thalassemia patients was found to be 192.9 μg/dl (range 63-602 μg/dl) (normal 60-150). Only six patients had serum iron levels above 300. Serum ferritin levels ranged between 1100-6300 ng/ml (average 2341.98 ng/ ml). Twenty-eight (62%) patients had serum ferritin levels between 1500-3000 ng/ml.

Of the 19 patients who received desferrioxamine therapy, 11 had lenticular opacities. However, only seven of 26 (27%) patients not on desferrioxamine had lenticular opacities. Thus, lens opacities were significantly more in patients receiving desferrioxamine (*P* < 0.05). Prolonged exposure to desferrioxamine therapy too led to increased occurrence and density of lens opacities. Those with no lens opacities had taken desferrioxamine on an average for 1.94 years. Of the 33 patients who were on deferriprone therapy, 14 (42%) had lenticular opacities while four of 12 subjects not on deferriprone therapy had lens opacities. In Group C, patients with no lens opacities had taken an average deferriprone dose of 2701 g for three years which was higher than those with lens opacities in that group. Similar observations were recorded in Group D where patients with no lens opacities had taken an average deferriprone dose of 2736.3 g for 3.5 years. However, no significant correlation was found between occurrence of lens opacities and deferriprone therapy. In those without lens opacities, average serum iron levels, average serum ferritin levels and average number of blood transfusions received were 140.07 mcg/dl, 1766.18 ng/ ml and 120.25 respectively while in those with lens opacities, they were 257.2, 3075.5 and 252 respectively. Correlation of serum iron, serum ferritin and number of blood transfusions with presence of lens opacities was found to be statistically significant (*P* < 0.001).

Nine of 25 (36%) males and nine of 20 (45%) females had lens opacities. The difference was found to be statistically significant. RPE degeneration, RPE mottling and retinal vessel tortuosity, all were found less in patients receiving higher dose of desferrioxamine therapy (Group B) [Table 4]. The opposite was found in those receiving higher dose of deferriprone therapy. In those with decreased visual acuity in Group D, the average deferriprone dose received was found to be higher. Table 5 shows the correlation of average serum iron levels, serum ferritin levels and number of blood transfusions in various groups with fundus and visual acuity changes. In those with RPE degeneration, RPE mottling and visual acuity changes, serum iron levels, serum ferritin levels and number of blood transfusions received all were higher than in those without these changes. Same was the case with retinal vessel tortuosity except in Group B where tortuosity was more in patients who had received lesser number of transfusions. Disc hyperemia too was more in patients with higher serum iron and ferritin levels and in those who had received larger number of transfusions. The average age of patients with disc hyperemia was 14.66 years. Regular follow-up of all patients for one year did not reveal any change in ocular status.

**Table 4:** Fundus and visual acuity changes in desferrioxamine and deferriprone groups

	Desferrioxamine therapy (Group B)			Deferriprone therapy (Group D)		
		
	No. of subjects (n = 6)	Average dose of therapy (in g)	Average duration of therapy (in years)	No. of subjects (n = 20)	Average dose of therapy (in g)	Average duration of therapy (in years)
RPE* degeneration						
Present	1	416	2	9	4065.69	4.4
Absent	5	426.4	2.6	11	1870.61	3
RPE mottling						
Present	0	-	-	2	4106.25	5
Absent	6	424.6	2.5	18	2719.75	3.5
Retinal vessel tortuosity						
Present	1	416	2	2	5110	5.5
Absent	5	426.4	2.6	18	2608.22	3.44
Visual acuity						
Normal	3	517.33	3	14	2356.2	3.42
Decreased	3	312	2	6	4030.2	4.16

* -Retinal pigment epithelium

**Table 5:** Correlation of average serum iron levels, serum ferritin levels and number of blood transfusions in subjects with fundus and visual acuity changes in various groups

	Group A		Group B		Group C		Group D	
				
	Present	Absent	Present	Absent	Present	Absent	Present	Absent
RPE* degeneration								
Avg. serum iron (mg/dl)	-	171.5	250	162.4	369.5	177.6	195.2	158.2
Avg. serum ferritin (ng/ml)	-	2025.8	2664	2102	4513	2049.3	2489.8	1923.8
Avg. blood transfusions	-	51.8	234	145.8	351.75	189.2	229.3	136
RPE mottling								
Avg. serum iron (mg/dl)	602	85.4	-	177	280	233.7	251	166.4
Avg. serum ferritin (ng/ml)	6300	1171	-	2195	4568	2592.3	3414.5	2063.4
Avg. blood transfusions	240	14.2	-	160.5	401	227.1	310	160.8
Retinal venous tortuosity								
Avg. serum iron (mg/dl)	602	85.4	250	162.4	280	233.8	262.5	165.1
Avg. serum ferritin (ng/ml)	6300	1171	2664	2102	4568	2592.3	3949.5	2009.1
Avg. blood transfusions	240	14.2	23.4	145.8	401	227.1	251.5	166.5
Visual acuity changes								
Avg. serum iron (mg/dl)	602	85.4	184.7	169.3	328.2	180.5	200.5	163.8
Avg. serum ferritin (ng/ml)	6300	1171	2264.7	2126.7	4234.2	191.6	2384.6	2090.1
Avg. blood transfusions	240	14.2	199	122	343	177.4	254.7	141.9

* -Retinal pigment epithelium, Avg.-Average

## Discussion

Patients suffering from thalassemia present with varied ocular and systemic manifestations. Ocular findings range from decreased visual acuity, color vision anomalies and night blindness to cataract, visual field defects and optic neuropathy. Iron-chelating agents like desferrioxamine and deferriprone too are reported to cause many of these ocular changes. Studies of ocular changes in thalassemics from the Indian subcontinent are very few. In our study, we studied the various ocular manifestations of thalassemia and also the effects of various iron-chelating agents on the eye.

In our study, patients belonged to the age group six months to 21 years. However, similar studies in the Western world have also been performed in patients of up to 45 years of age. This age disparity can be attributed to lower survival rates among thalassemics in the Indian subcontinent, unlike the West. Reasons for this seem to be poor compliance with therapy, difficulty in obtaining regular blood transfusions and high cost of iron chelation therapy. The literature provides no clue as to whether thalassemia is more preponderant in a particular sex. In our study, a slight male preponderance (1.25:1) was observed over females. This is consistent with the studies of Gartaganis *et al*.[12] and Gaba *et al*.[13] where ratios of 1.07:1 and 1.33:1 respectively, were observed.

Frequency of ocular involvement in our study was 58%. Gartaganis *et al*. reported figures of 41.3% while Gaba *et al*. reported ocular involvement in 71.4% of subjects in their respective studies. Our figure of 58% is in between those of the above studies. The Indian subcontinent and the Middle East are known to be high-prevalence areas of beta-thalassemia. Our study had a large number of subjects originally hailing from Pakistan (44%) which is consistent with the known geographical distribution of the disease. Age at diagnosis is directly related to the time at which anemia manifests itself. In our study, the maximum number (36/45 = 80%) of patients were diagnosed within the first year of life. In the study conducted by Gaba *et al*., 55% patients were diagnosed in the first year of life. An earlier detection rate in our study can be attributed to better and cheaper diagnostic facilities extended by our Government as well as mass media coverage about the disease which helps in spreading awareness in the society. Blood transfusions in beta-thalassemia major aim to maintain the level of hemoglobin at 10-14 g/dl. Average number of blood transfusions in our study was 176.15. In Gaba *et al*.'s study, the average number of transfusions received were 142. Eighteen out of 45 subjects in our study had lenticular opacities. Gartaganis *et al*. and Gaba *et al*., in their respective studies, found lens opacities in 13.8% and 45.7% of subjects. None of these opacities were in the visual axis and none therefore interfered with vision. Lens opacities were more common in females (9/20), (45%) than in males (9/25), 36%. However, the difference was not statistically significant. Gaba *et al*. too reported a higher incidence of lens opacities in females in their study (*P* = 0.037). In our study, lens opacities correlated significantly with higher average serum iron levels, ferritin levels and number of blood transfusions received (*P* < 0.001). This is consistent with the findings of Gaba *et al*. Iron-chelating agents too have been implicated in the causation of lens opacities. Gartaganis *et al*., in their study, found no correlation between occurrence of lens opacities and dose of desferrioxamine received. The opposite was found in Gaba's *et al*. study (*P* < 0.05). Our study results are consistent with those of Gaba *et al*. (*P* < 0.05). Incidence of lens opacities was higher in Group B as compared to Group C, thereby indicating the superiority of combined iron-chelating therapy in terms of lower individual doses of drugs required.

In our study, unaided visual acuity was found normal in 30 (67%) patients while in Gaba *et al*.'s study, the figure was 62.9%. Recent studies conducted by Taher *et al*.[14] in 2006 have found normal visual acuity in 80.6% subjects. Taher *et al*. also found that the type of iron-chelating agent used had no influence on decrease in visual acuity. This observation is consistent with the findings of our study. We found RPE degeneration in 31% patients, more with increasing age. Gartaganis *et al*. and Gaba *et al*. reported figures of 37.5% and 31.4% respectively in their studies. Statistical significance was found between RPE degeneration and higher average serum iron levels, serum ferritin levels and number of blood transfusions received. Our study showed that patients with RPE changes had received lesser doses of desferrioxamine and larger doses of deferriprone thus indicating that desferrioxamine may be protective while deferriprone use may be contributory to the occurrence of RPE degeneration. The findings are consistent with those of Taher *et al*. who had found patients on deferriprone to be four times more likely to have RPE degeneration as compared to patients on desferrioxamine. However, these differences were not statistically significant. RPE mottling was seen in 9% patients in our study, mostly in higher age groups. Correlation of serum iron levels, serum ferritin levels and number of blood transfusions with RPE mottling was statistically significant. None of the patients in Group B had RPE mottling which points to a protective effect exerted by desferrioxamine on RPE mottling. Similar observations were reported by Gaba *et al*. RPE mottling was more in patients receiving deferriprone therapy although the difference was not statistically significant (*P* = 0.369). Retinal venous tortuosity was observed in 11% of patients in our study. This is similar to the incidence reported by Gaba *et al*. (17.14%) and Taher *et al*. (17.9%). Here too, tortuosity was more in patients with higher serum iron and ferritin levels and those who had received more blood transfusions. Gaba *et al*. have also reported similar observations.

The solitary patient in Group B having venous tortuosity was receiving lesser doses of desferrioxamine. Although not much inference can be drawn from this, it is the contention of the authors that poor chelation may be an important cause of vessel abnormality. This is in contrast to the observations of Gaba *et al*. who reported significantly higher retinal venous tortuosity in patients on iron chelation therapy. However, Gaba *et al*.'s patients had received desferrioxamine intravenously (rather than the extremely effective subcutaneous route) whereby the ability of the drug to cause iron chelation is suspect.

Follow-up of patients quarterly for one year did not reveal any change in ocular status. A longer follow-up period is required to analyze and comment on how ocular changes may evolve. A limitation of the present study is that it cannot conclusively establish whether ocular changes are a result of the disease per se or due to iron-chelating agents. This requires stoppage of chelation therapy. It may be kept in mind though that iron overload and iron-chelating agents both may be mutually confounding factors in the causation of ocular changes of thalassemia. Also, patients enrolled in our study were already following a fixed thalassemia treatment regimen from elsewhere. In order to study the ocular manifestations of thalassemia in these patients and the ocular side-effects of iron-chelating agents, it was imperative that the treatment regimes being followed at presentation be continued. In other words, group changes were not allowed by way of randomization for the sake of prospectively observing the ocular effects of each treatment regime. A study on newly diagnosed cases of thalassemia not on any treatment as well as a long-term follow-up of such patients in order to ascertain the development and evolution of ocular changes is suggested.

## Conclusion

Most of the ocular changes of beta-thalassemia are attributed to the course and severity of the disease. Reduction in serum iron and ferritin levels by iron-chelating agents and regular ocular examination to look for side-effects of such agents can aid in preventing, delaying or ameliorating ocular complications.
